# Anthelmintic resistance and prevalence of gastrointestinal nematodes infecting sheep in Limpopo Province, South Africa

**DOI:** 10.14202/vetworld.2021.302-313

**Published:** 2021-02-02

**Authors:** Morutse Mphahlele, Ana M. Tsotetsi-Khambule, Rebone Moerane, Dennis M. Komape, Oriel M. M. Thekisoe

**Affiliations:** 1 Unit for Environmental Sciences and Management, North-West University, Potchefstroom, 2531, South Africa; 2 Department of Life and Consumer Sciences, College of Agriculture and Environmental Sciences, University of South Africa, Florida, 1709, South Africa; 3 Department of Epidemiology, Parasites and Vectors Programme, ARC-Onderstepoort Veterinary Research, Onderstepoort, 0110, South Africa; 4 Production Animal Studies, Faculty of Veterinary Science, University of Pretoria, Onderstepoort, 0110, South Africa

**Keywords:** anthelmintic resistance, fecal egg counts, FAMACHA, seasonal prevalence, sheep

## Abstract

**Background and Aim:**

Previous studies recorded the prevalence of gastrointestinal nematodes (GIN) in Limpopo Province. However, the studies did not address the seasonal patterns of infection and did not cover all districts of Limpopo Province, namely; Capricorn, Sekhukhune, Waterberg, Mopani, and Vhembe. It is, therefore, important to provide up to date information on the prevalence and seasonal occurrence data of GIN in all districts of Limpopo province. The present study was conducted to determine the occurrence of anthelmintic resistance (AR) and document the prevalence of GIN infecting sheep in five districts of Limpopo Province, South Africa.

**Materials and Methods:**

Forty animals in each district were used for fecal egg count reduction test (FECRT) to determine AR against ivermectin (0.2 mg/kg), levamisole (LEV) (5 mg/kg), and albendazole (7.5 mg/kg). Egg hatch test (EHT) was used to determine AR against thiabendazole (TBZ) and micro-agar larval development test (MALDT) was used for both TBZ and LEV. Naturally, infected sheep (n=780) were sampled for prevalence across five districts of Limpopo. FAMACHA^©^ eye-color score estimations were also performed for each study animal.

**Results:**

FECRT showed occurrence of AR in most of the districts and a few with suspected resistance. EHT results showed AR development against TBZ for all districts, while the MALDT showed no AR against LEV in all districts, but detected AR against TBZ in Sekhukhune, Capricorn, and Waterberg. *Haemonchus contortus* was the most resistant species. A high nematode prevalence (88-100%) and 1210-1861 eggs per gram (EPG) was observed in all districts during the hot wet season, decreasing to 75-80% (453-1202 EPG) during the cold dry season. The sheep revealed a FAMACHA^©^ mean score of 3, indicating mild anemia during the hot wet season except for Vhembe district that revealed a FAMACHA^©^ mean score of 4 during the hot wet season, indicating anemia.

**Conclusion:**

AR recorded in Limpopo Province may be due to under-dosing caused by lack of weighing equipment and high treatment frequencies due to lack of proper training on anthelmintic use. The detection of AR in Limpopo is an important finding because it will help in outlining effective management systems against GIN.

## Introduction

Gastrointestinal nematodes (GIN) are responsible for substantial losses in the animal production industry [1]. Anthelmintics have been used as the primary control measure for nematode parasites in sheep [2]. However, over the years, there has been continuous and significant development of anthelmintic resistance (AR) by the parasitic worms infecting livestock [3]. AR can be defined as the ability of parasites to survive doses of drugs that would normally kill parasites of the same species and stage. AR is inherited and selected for because the survivors of treatments pass genes for resistance onto their offspring [4]. AR has been reported for all anthelmintic classes currently available, namely, benzimidazoles (BZ) (e.g., flubendazole, albendazole, and fenbendazole), imidazothiazoles (e.g., levamisole [LEV]), and macrocyclic lactones (ML) (e.g., ivermectin) [5]. In South Africa, AR has been reported in sheep [6,7] and goats [7-9] in both the commercial and resource-poor farming systems. The overall prevalence of AR in South Africa and elsewhere in Africa has, however, not been extensively investigated.

Research conducted in many parts of the world, particularly in countries such as the UK, Australia, South Africa, and New Zealand, has resulted in a growing awareness of the challenges brought about by AR, but also recognition of potential resistance-delaying strategies, which can be used on farms [10]. The three main factors for the selection for AR are high treatment frequency [11], under-dosing and management such as poor pasture management can accelerate the development of AR [12]. Anthelmintic drugs currently used in South Africa include BZ (Valbazen^®^, Zoetis United States), ML (Ivomec^®^, Merial, United States), LEV (Tramisol Ultra^®^, Coopers and Intervet), and praziquantel + LEV + oxfendazole + abamectin combination (Triple A plus, Virbac, New Zealand). The pathogenic implications of GIN infections on host welfare are clear [13]. It is, therefore, crucial to monitor the prevalence and distribution of livestock helminth species to plan sustainable control, especially because prevalence studies may become rapidly outdated and there is no structured mechanism in place to monitor livestock helminth prevalence over time [14,15]. Moreover, environmental conditions, particularly temperature and humidity, affect the distribution of species and the presence of parasites. Seasonal variations also play an important role in prevalence of GIN. As such, changes in humidity and temperature can influence development, survival, and transmission of parasites in the external environment because they provide favorable environmental conditions for transmission of GIN [1].

The aim of this study was to determine the occurrence of AR and document the prevalence of GIN of sheep in selected flocks in all districts Limpopo Province, South Africa.

## Materials and Methods

### Ethical approval

The study was approved by the scientific committee of Integrated Pest Management, North-West University, with reference no: NWU-01252-19-A9 and Department of Agriculture, Forestry and Fisheries (DAFF) in terms of section 20 of the Animal Diseases Act (35 of 1984). Participating resource-poor farmers granted permission to collect study samples from their sheep.

### Study area, period and animal management

**Figure-1:**
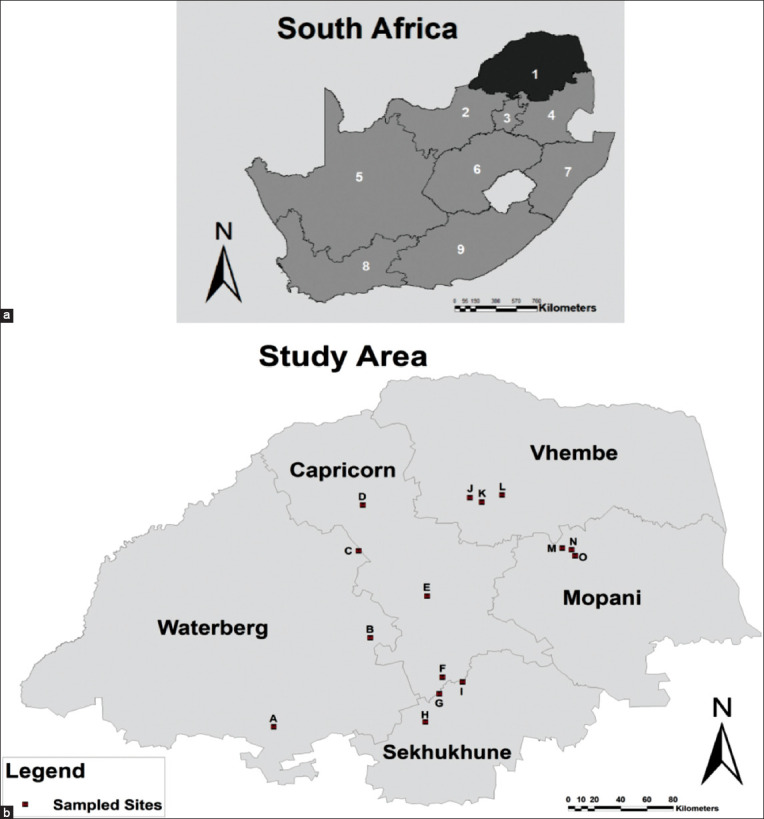
(a) Map showing nine provinces of South Africa with Limpopo Province shown in a dark grey color. (b) Locality map depicting study sites from Limpopo districts for seasonal prevalence of gastrointestinal nematodes: (A) Bela-Bela (B) Mokopane and (C) Ga-Ramela village from Waterberg district; (D) Ga-Kobe village, (E) Makotse village, and (F) Tooseng village from Capricorn district; (G) Malope village, (H) Tompi Seleka College, and (I) Strydkraal village from Sekhukhune district; (J) Madodonga village, (K) Ha-Ramantsha village and (L) Louis Trichardt from Vhembe district; (M) Ga-Maupa village, (N) Mamokgadi village and (O) Mamatlepa village from Mopani district. Villages circled in red show where anthelmintic resistance studies were conducted.

The study was conducted on small-scale farming locations in five districts of Limpopo Province, namely, Capricorn, Sekhukhune, Waterberg, Mopani, and Vhembe (Figure-1). The sheep in these locations were managed under an extensive system where communal grazing was shared among the resource-poor farmers. Meteorological data for the collection sites starting from January 2017 to November 2018 were obtained from South African Weather Service. The data included weather elements such as temperature, rainfall, and humidity.

For the selection of animals to be included in the AR part of the study, the resource-poor farmers provided information regarding anthelmintic classes they use in their flocks, how often they treat their livestock, how often they change the active ingredients, and whether they weigh their animals before treatment with anthelmintics. Experimental animals for fecal egg count reduction test (FECRT) were selected from the flocks that had a long history of anthelmintic treatment and high treatment frequencies. Five flocks with the highest treatment frequencies were selected from the 77 flocks that were registered in the extension officer’s database. In each district, 40 animals belonging to one farm were divided into groups of ten animals per group. Furthermore, only animals that had not been treated for GIN for at least 8 weeks before sampling were selected. Sheep were reared under extensive grazing systems whereby they were released during the day to graze on communal lands and kept in holding pens at night.

To determine prevalence of GIN, 468 fecal samples were collected on a bi-monthly basis between March 2017 and January 2018 for Capricorn, Sekhukhune, and Waterberg districts. A further 312 samples were collected between January and November 2018 for Mopani and Vhembe districts. All in all, a total of 780 naturally infected sheep comprising of Pedi sheep and Pedi × Dorper cross breeds were sampled. The average weight of the study animals (1-2 years old) was 29.5 kg and 35.2 kg for females and males, respectively. The animals were ear tagged so that samples could be taken from the same animals throughout the duration of the study period. FAMACHA^©^ eye-color score estimations [16,17] were also performed for each study animal.

### Fecal collection and coprological examination

Fecal samples were collected directly from the rectum of sheep into clean, labeled plastic bags and stored in a cooler box at 2-4°C. They were transported immediately to the Helminthology Laboratory at the Agricultural Research Council, Onderstepoort Veterinary Institute for coprological examination. The fecal samples were examined for the presence of GIN eggs; nematode egg counts were made using the McMaster technique with minimum detection limit of 100 EPG [18]. Briefly, 2 g of feces was mixed thoroughly with 58 mL of 40% sugar solution ensuring that the fecal pellets were disrupted. A Pasteur pipette was used to transfer aliquots of the mixture into the two chambers of a McMaster slide. The slide was left to stand for 5-10 min to allow the eggs to float to the surface of the medium. The slide was examined using a dissecting microscope (Nikon Eclipse E100 Company, Japan) at 10× and/or 40×. Nematode eggs were counted in each chamber of the McMaster slide and the EPG was calculated using the following formula


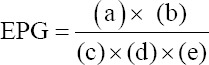


where (a) = (total number of eggs counted);

(b) = 60 (total volume of fecal suspension);

(c) = 2 (the number of chambers counted);

(d) = 2 (grams of feces);

(e) = 0.15 mL (standard volume of the chamber).

### *In vivo* assays

#### FECRT

The FECRT as described by Coles *et al*. [19] was used to determine the presence of AR. The minimum egg count for inclusion in the pre-treatment group, excluding the control group, was 1,000 EPG. Group 1 was treated subcutaneously with ivermectin (Ivomec^®^, Merial, United States, 0.2 mg/kg bw), Group 2 was orally dosed with LEV (Tramisol Ultra^®^, Coopers and Intervet, New Zealand, 5 mg/kg bw), and the Group 3 was orally dosed with albendazole (Valbazen^®^, Pfizer, United States, 7.5 mg/kg bw). Group 4 represented the untreated control. Most of the nematode eggs are morphologically indistinguishable; hence, cultures were prepared from feces collected both before and after treatment to identify nematode genera as described by Van Wyk and Mayhew [20].

### *In vitro* assays

#### Egg hatch test

The egg hatch test and Micro-argar larval development test (MALDT) were performed as described by Coles *et al*. [19] for the *in vitro* determination of BZ and LEV resistance, respectively. For both assays, nematode eggs were recovered from the same fecal samples that were collected before treating the animals in the FECRT using the nematode egg recovery method as described by Maphosa *et al*. [21] with some minor modifications.

For egg hatch test (EHT), *ca*. 100 nematode eggs were pipetted into a 96-well microtiter plate and then 10 μL of thiabendezole (TBZ) solution added [22]. The final concentrations of TBZ were 0.05, 0.1, 0.2, 0.3, and 0.5 μg/mL dissolved in dimethyl sulfoxide (DMSO). In addition, a negative control (distilled water) was tested. All tests were duplicated. The plates were covered and incubated under humidified conditions for 48 h at 27°C. A drop of Lugol’s iodine solution was added to each well to stop further hatching and the number of unhatched eggs and the first stage larvae (L_1_) present per well were counted. Inhibition percentages were calculated using a formula described by Cala *et al*. [23].


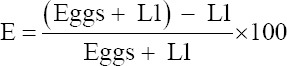


The discriminating dose (DD) for the EHT is a dose that prevents the hatching of 99% of susceptible eggs; all the eggs that hatch are resistant [19]. The level of resistance was determined by the number of eggs hatching in the DDs of 0.1 μg/mL TBZ. The percentage of hatched eggs is thus a direct measure of BZ resistance.

### MALDT

For the MALDT, stock solutions of TBZ/LEV were prepared by pre-dissolving the drugs in DMSO with subsequent dilution in distilled water (1:4). One hundred nematode eggs were incubated for 7 days at 27°C in 96-well microtiter plates with culture medium (yeast extract with Earle’s Balanced Salt Solution and physiologic salt solution) in an aquatic solution of various concentrations (range from 0.0006 to 1.28 μg/mL) of thiabendazole (TBZ)/LEV [19]. After 7 days, the numbers of unhatched eggs and L_1_ – L_3_ larvae in each well were counted under an inverted microscope. The rate of L_3_ development in the DD (0.02 μg/mL and 0.5 μg/mL for TBZ and LEV, respectively) compared to the control was used to determine if resistance was present. The number of larvae developing from L_1_ to L_3_ stage in the DD of 0.02 μg/mL TBZ and 0.5 μg/mL LEV was an indication of resistance. The test was performed in two replicates for each drug concentration.

Prevalence was determined using the following formula [24]:


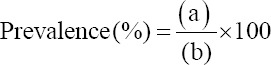


Where, “a” = Number of individuals having a disease at a particular time; “b” = Number of individuals in the population at risk at that point in time. Fecal cultures were prepared to identify nematode genera [20] and determine distribution of nematode genera in Limpopo Province. The L_3_ of *Trichostrongylus* spp. of small ruminants are difficult to differentiate from those of *Teladorsagia* spp. because they are similar in length and can only be differentiated generically by tail morphology after exsheathment. Consequently, they were grouped during the count.

### Statistical analysis

The FECRT (FECRT %) was calculated using the formula of Kochapakdee *et al*. [25]. FECRT % = 100 × (1−(T2/T1), where T2 represents FEC post-treatment and T1 represent FEC pre-treatment [25]). SAS Statistics (Version 9.4) was used to analyze FECRT data for confidence limits and the Pearson’s correlation coefficient (Pearson’s r) was used to measure the linear correlation between variables.

Resistance was said to be present if the FECRT % was less than 95% and the lower limit of the 95% confidence interval was less than 90%. If only one condition was met, then resistance was only suspected [8,19,26].

Instead of using the traditional threshold values (LC 50 or LC 99), the threshold discriminating concentrations were used for both EHT and MALDT because it is faster, simpler, and cheaper [27]. Furthermore, the LC 50 criterion is not able to provide early detection during the development of resistance [28].

## Results

Using the analysis described by Kochapakdee *et al*. [25], the results revealed development of AR against all the anthelmintic classes with percentages of ≤95% and ≤90% lower confidence limit (LCL) in all the districts of Limpopo Province except for Sekhukhune, where AR was suspected against all the three anthelmintic classes (Table-1). Furthermore, AR was also suspected against ML in Capricorn and LEV in Vhembe districts flocks at 92% FECRT with LCL of 90% and 90% FECRT with a 78% LCL, respectively. However, there was no significant difference (p>0.05) in the FECRT results between all the three anthelmintic classes tested.

**Table-1:** Fecal egg count reduction test and lower limits of 95% confidence level calculated on the basis of individual animal’s egg counts before and after treatment on the same sheep using method of Kochapakdee *et al*. [27]. FECRT%=100 × (1−(T2/T1).

Study site	Anthelmintic used	FEC1 (Range)	FEC2 (Range)	FECRT%	Lower limit of 95% confidence	Interpretation of results
Sekh	BZ	1940 (1455-2425)	80 (60-100)	96	93	Suspected resistance
	LEV	2080 (1560-2600)	150 (112-187)	94	90	Suspected resistance
	ML	2030 (1522-2537)	120 (90-150)	96	90	Suspected resistance
	Control	2080 (1560-2200)	2060 (1840-2560)	-	-	-
Capr	BZ	1790 (895-3580)	200 (100-400)	92	85	Resistant
	LEV	1660 (830-3320)	130 (65-260)	93	88	Resistant
	ML	1840 (920-3680)	120 (60-240)	92	90	Suspected resistance
	Control	1750 (1430-2080)	1940 (1850-2300)	-	-	-
W/berg	BZ	2840 (2200-3500)	1300 (400-2300)	56	40	Resistant
	LEV	2960 (1300-5000)	580 (200-900)	79	72	Resistant
	ML	4440 (2200-7200)	1340 (0-2000)	65	50	Resistant
	Control	1570 (1410-1600)	1580 (1380-1670)	-	-	-
Mopani	BZ	1960 (500-5000)	620 (300-880)	47	26	Resistant
	LEV	1620 (400-2300)	480 (100-900)	70	61	Resistant
	ML	1280 (500-2400)	320 (100-900)	72	51	Resistant
	Control	1180 (850-1250)	1180 (900-1250)	-	-	-
Vhembe	BZ	2660 (500-4400)	340 (0-1000)	89	82	Resistant
	LEV	2525 (400-7800)	50 (0-500)	90	78	Suspected resistance
	ML	1060 (500-2000)	160 (0-300)	90	80	Resistant
	Control	1290 (1100-1350)	1280 (1080-1580)	-	-	-

Sekh=Sekhukhune, Capr=Capricorn, W/berg=Waterberg, BZ=Benzimidazole (Valbazen^®^), LEV=Levamisole (Tramisol Ultra^®^), ML=Macrocyclic lactones (Ivomec^®^), FEC1=Fecal egg count pre-treatment, FEC2=Fecal egg count 14 days post-treatment

**Figure-2:**
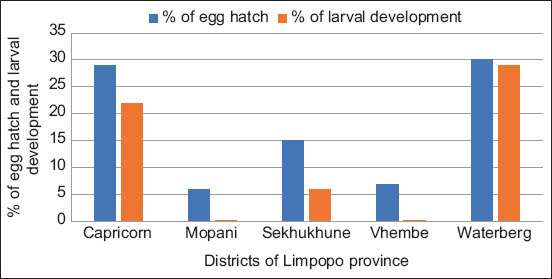
The percentage of eggs that hatched at a discriminating dose (DD) of 0.1 µg/mL thiabendazole and eggs that developed to third stage (infective) larvae in the DD of thiabendezole (0.02 μg/mL) in the macro-agar larval development test.

The EHT results showed that there was resistance against TBZ, which is a BZ, because it had a minimal ovicidal effect that resulted in more than 1% egg hatchability on nematode eggs at a DD of 0.1 μg/mL in all 5 (100%) flocks that were investigated in Limpopo. Figure-2 shows the hatchability percentages at the DD of 0.1 μg/mL TBZ. In the negative controls (water), the percentage of eggs hatching was >95% for all flocks.

The MALDT showed no AR against LEV as 100% larval development inhibition was recorded in all the investigated flocks at 0.5 μg/mL DD. However, AR was detected against TBZ as development of the larvae from L_1_ to L_3_ was recorded in Sekhukhune, Capricorn, and Waterberg flocks at 6, 22, and 28%, respectively, at 0.02 μg/mL DD (Figure-2).

Table-2 shows that the fecal examination test for the presence of nematode eggs in the pre-treatment fecal samples revealed that all sheep (100%) were positive for strongyle eggs. Nematode genera/species that were identified pre-treatment from the larval cultures included *Haemonchus contortus*, *Teladorsagia/Trichostrongylus*, and *Oesophagostomum columbianum*. The *H. contortus* was identified as the most frequently encountered resistant nematode post-treatment in all five flocks. On the other hand, among all the nematode genera identified pre-treatment, *O. columbianum* was the only species that was not detected post-treatment in all the anthelmintic treatments (Table-2).

**Table-2:** Results of the percentage of gastrointestinal nematode genera/species identified from the larval cultures at day 0 pre-treatment and day 14 post-treatment.

Anthelmintic group	*Haemonchus contortus*		*Tel/Trich* spp.		*Oesophagostomum columbianum*	
Sekhukhune	D 0	D 14	D 0	D 14	D 0	D 14
BZ	74	90	23	10	3	0
LEV	75	75	20	25	5	0
ML	87	96	10	4	3	0
Control	83	84	15	16	2	0
Capricorn						
BZ	93	97	4	3	3	0
LEV	87	93	10	7	3	0
ML	88	93	10	7	2	0
Control	82	86	15	14	3	0
Waterberg						
BZ	89	100	9	0	2	0
LEV	83	94	11	0	6	0
ML	85	100	10	0	5	0
Control	91	92	7	8	2	0
Mopani						
BZ	88	100	12	0	0	0
LEV	87	100	13	0	0	0
ML	95	100	5	0	0	0
Control	95	92	4	5	1	1
Vhembe						
BZ	90	100	8	0	2	0
LEV	90	100	5	0	5	0
ML	92	100	7	0	1	0
Control	93	92	6	4	1	2

*Tel=Teladorsagia, Trich=Trichostrongylus*, BZ=Benzimidazole, LEV=Levamisole, ML=Macrocytic lactones, D0=Treatment day 0, D14=Treatment day 14

**Figure-3:**
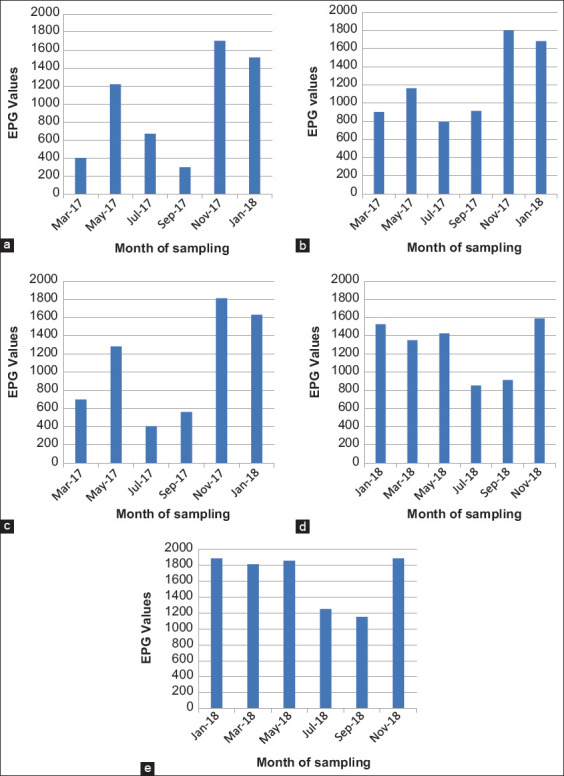
Seasonal incidence of gastrointestinal nematodes of sheep in Limpopo (a) Capricorn district, (b) Sekhukhune district, (c), Waterberg district, (d) Mopani district, and (d) Vhembe district.

Distribution of GIN in Limpopo Province was continuous throughout the year with the highest EPGs recorded during the hot wet season between November and January, with the highest peak observed in Vhembe district in November 2018 (EPG 1887) and in Waterberg district in November 2017 (EPG 1811). On the contrary, the lowest EPGs during the cold dry season were recorded between July and September, with the lowest EPG counts recorded for Capricorn district (EPG 299) in September 2017 (Figure-3).

**Figure-4:**
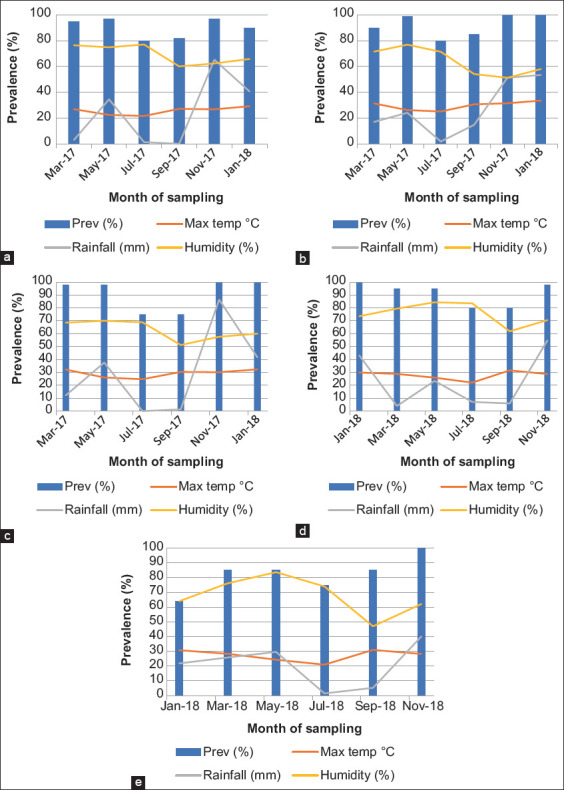
The relationship between rainfall, humidity, and maximum temperature on the prevalence of gastrointestinal nematodes per month/season in Limpopo (a) Capricorn district, (b) Sekhukhune district, (c), Waterberg district, (d) Mopani district, and (e) Vhembe district.

Figure-4 details a high nematode prevalence ranging from 64% to 100% during the hot wet season and a decrease to a range of 75-80% prevalence during the cold dry season. However, there was no significant difference (p>0.05) in the mean EPGs between the different seasons and so was for the nematode prevalence in different seasons.

**Figure-5:**
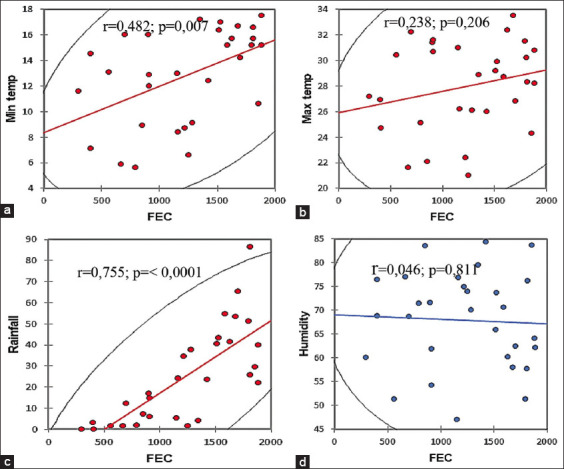
Correlation between minimum temperature and fecal egg counts (a), maximum temperature and fecal egg counts (b), rainfall and fecal egg counts (c), and humidity and fecal egg counts (d) in the five districts of Limpopo Province.

Furthermore, a high nematode prevalence correlated with the high rainfall experienced during November in all the districts, being particularly high in Waterberg and Capricorn districts with 68.4 mm and 65.2 mm, respectively (Figure-4). Figure-5 shows a positive correlation between mean minimum temperature and mean FEC (r=0.482; p=0.007) and between mean rainfall and mean FEC (r=0.755; p=≤0. 0001) in all the districts of Limpopo.

However, an overall poor correlation existed between mean humidity and mean FEC (r=0.046; p=0.811) in all the districts. In addition, the sheep examined in Vhembe district during the hot wet season revealed a FAMACHA^©^ mean score of 4, indicating anemia. In all other districts, the sheep revealed a mean score of 3, indicating mild anemia, during the hot wet season (Table-3).

**Table-3:** Mean maximum temperature, rainfall, FEC, and FAMACHA^©^ scores during hot wet and cold dry seasons.

District	Maximum temp		Rainfall (mm)		FEC		FAMACHA^©^ Score	
			
	Hot wet	Cold dry	Hot wet	Cold dry	Hot wet	Cold dry	Hot wet	Cold dry
Capricorn	26.3^a^	24.4^a^	35.8^a^	0.7^a^	1210^a^	485^a^	3^a^	2^a^
Sekhukhune	27.9^a^	30.6^a^	36.4^a^	8.3^a^	1386^a^	853^a^	3^a^	2^a^
Waterberg	30.0^a^	27.5^a^	44.4^a^	0.7^a^	1356^a^	483^a^	3^a^	1^a^
Mopani	28.3^a^	26.8^a^	31.3^a^	6.6^a^	1473^a^	882^b^	3^a^	2^b^
Vhembe	28.0^a^	26.0^a^	29.3^a^	3.3^b^	1861^a^	1202^b^	4^a^	2^b^

Means with the same letter (^a-b^) are not significantly different (p=0.05). FEC=Fecal egg count

In general, there was a strong correlation (r=0.959; p=≤0. 0001) between FEC and clinical anemia in sheep. On the other hand, the sheep examined in all the districts during the cold dry season were non-anemic, as indicated by the FAMACHA^©^ score of 1 for sheep in the Waterberg and a score of 2 for the animals in all the other districts. Higher EPGs and FAMACHA^©^ scores were observed during the hot wet season, while the opposite was seen during the cold dry season, which indicated that worm burden was responsible for anemia in sheep (Table-3).

**Figure-6:**
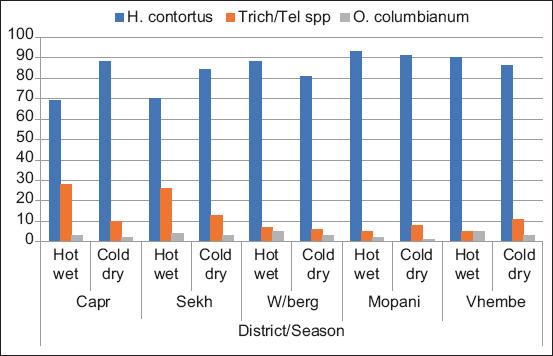
Mean percentage of gastrointestinal nematode genera in five districts of Limpopo Province during different seasons of the year.

The results of the composite fecal cultures study revealed the highest prevalence of the very prolific worm *H. contortus*. Figure-6 shows the mean percentage of GIN genera in the five districts of Limpopo Province during hot wet and cold dry season. The *H. contortus* was the dominant nematode species, representing 70-93% of the total larval recovery throughout the entire study period in all the districts of Limpopo Province, with the highest percentage of 93% recorded for Mopani in November 2018.

*Trichostrongylus/Teladorsagia* spp. were the next most prevalent parasites, which represented 5-28% of the total infective larvae harvested from the composite fecal cultures during the study period. The *O. columbianum* was detected in low percentages, never exceeding 5% throughout the study period.

## Discussion

Based on the results of the FECRT, resistance to BZ was detected in all the flocks except for those in the Sekhukhune district. Resistance was also confirmed by the hatching of eggs in the *in vitro* egg hatch test (EHT), which is regarded as highly sensitive [29]. An overwhelming majority of farmers, ranging from 45% in Mopani to 95% in Sekhukhune districts, used BZ to treat GIN of sheep in their flocks and many never changed the anthelmintic class. Similar results were obtained in the southern part of Ethiopia where albendazole was the most commonly used anthelmintic drug used by resource-poor farmers [30].

FECRT% of 56 and an LCL of 40% against BZ in a flock from Waterberg were obtained, which correlates with the highest number of hatching eggs (30%) and the highest number of larvae that developed (29%) as compared to all the other four flocks. The occurrence of resistance to BZ in Waterberg district was not unexpected since nine out of 11 farmers (82%) in that district never change the anthelmintic class, which in this case is BZ which was previously used in 55% of all the sampled flocks. The same could not be said for ML; however, because the FECRT results showed that there is resistance against this anthelmintic class even though it was the least used in all the districts of Limpopo Province, with no farmer in Sekhukhune and Waterberg ever mentioning its use in their flocks. This could be attributed to the fact that genes conferring AR are thought to be present in a small portion of individuals in the population even before the worms are exposed to a drug for the first time [12].

Earlier studies revealed that correlation between *in vivo* and *in vitro* tests is not always good [27]. Maharshi *et al*. [31] observed a poor correlation between FECRT results and EHT results for detecting BZ resistance. This is probably because *in vitro* tests are more sensitive than *in vivo* tests [28]. Even though some conflicting results were observed between the *in vitro* and *in vivo* assays in the present study, there are also outstanding cases where the results of both tests totally agree on flock resistance status. One such positive correlation is the non-development of larvae from L_1_ to L_3_ in the MALDT for detecting LEV resistance and high efficacy obtained from FECRT in Vhembe district flock. Field testing results indicate that *H. contortus* has remained generally susceptible to LEV for a longer period than to the other major drugs. LEV is used rarely as compared to BZ in Capricorn and Vhembe district flocks as it is to the rest of South Africa and many European countries [32]. It is against this backdrop that with proper use that includes administering the correct dosage for weight of the animal, pasture rotation, and other management tools, anthelmintics can still be used with maximum benefits [33].

The FECRT results obtained in the present study for the three tested anthelmintic classes coupled with EHT and MALDT results indicate occurrence of resistance in all the flocks except for Sekhukhune district flock where AR was suspected against all the anthelmintic classes. Another exception was for Vhembe district flock where AR was only suspected against LEV and ML in Capricorn. These results compare favorably with those of work done in Limpopo Province almost two decades previously, revealed LEV was <95% effective and BZ, only between 75 and 85% effective [6].

The predominance of *Haemonchus* spp. both pre- and post-treatment in the sheep found in the present study is consistent with the results obtained from studies conducted in Mpumalanga and Limpopo Province [6], North West Province [9], and Gauteng [7] where the most prevalent species was *H. contortus*. The reduction of *Trichostrongylus/Teladorsagia* spp. and *O. columbianum* and increase of *H. contortus* post-treatment could be attributed to high levels of pasture infectivity as a result of shorter generation interval of *H. contortus* which allows for greater contamination of pastures and re-infection of animals [34].

The seasonal dynamics of FEC in the present study followed the well-known pattern influenced primarily by temperature and moisture as they were highest in the hot-wet season and lowest in the cold-dry season [35].

The high prevalence of GIN seen in the current study could be due to the availability of suitable climatic conditions such as relatively high temperatures and rainfall that support the prolonged survival and development of an infective larval stage of most nematodes [36]. In the present study, *H. contortus* was the most prevalent nematode, followed by *Trichostrongylus/Teladorsagia* spp and *O. columbianum*. These results are comparable to those of earlier studies conducted in South Africa on the epidemiology of GIN that also reported *Haemonchus* spp. as the most dominant nematodes infecting sheep under commercial farming system [37] and under small-scale resource-poor farming conditions in South Africa [7,38]. These findings also corroborate well with those of studies conducted in Ethiopia and Brazil which showed *H. contortus* as the most prevalent nematode in small ruminants [39,40].

The observed results, of highest EPGs with the largest percentages of *H. contortus* L_3_ in fecal cultures during the rainy seasons, were in accordance with studies in other countries in Africa with distinct rainy and dry seasons, that is, Ghana [41], Kenya [42], and Zimbabwe [43]. This can be attributed to the high biotic potential of *H. contortus*, resulting in this parasite rapidly taking up dominance at times when environmental conditions on pasture are ideal for the development and survival of the free-living stages. The high biotic potential and shorter generation interval of *Haemonchus* spp. allow for greater contamination of pastures and re-infection of animals [34], which probably accounts for its higher prevalence than *Trichostrongylus/Teladorsagia* spp. and *O. columbianum* encountered in this study. In addition to the high biotic potential and shorter generation interval, a mature female of *Haemonchus* spp. can produce 5000-7000 eggs per day while *Trichostrongylus* spp. produce only 100-200 eggs per day; this shows a difference in their prolific reproduction abilities [44]. On the other hand, *Oesophagostomum* sp. has a very long generation interval of 45 days that cause its prevalence to be much lower [45].

Anemia observed in this study can be attributed to the GIN, predominated by *H. contortus* adult worms that cause harm as they cut to the mucosal lining in the abomasum, enabling them to suck blood from the mucosa of the GIT, causing blood loss, and development of anemia in sheep [46]. These observations agree with the results of previous studies where a relationship between EPG level and FAMACHA^©^ categories was elucidated and FAMACHA^©^ scoring was positively correlated with FEC levels in sheep [47,48]. However, the present study was in contrast with the study conducted by Mohammed *et al*. [49] that showed no correlation between EPG and FAMACHA^©^ score meaning that in their study, an increase in anemia did not necessarily result in an increased EPG which could have been due to mild infection in their study animals.

## Conclusion

The high prevalence of GIN in Limpopo Province could be due to conducive climatic conditions that support prolonged survival of infective ­larvae such as high temperatures and rainfall that prevailed in the province during the study period. High prevalence observed in this study translates to high numbers of animals with anemia and high nematode egg counts. It is for this reason that the FAMACHA^©^ system is recommended to accurately identify animals requiring anthelmintic treatment and consequently slow down the development of AR. The correlation of FAMACHA scores and FEC makes it a necessity to train resource-poor sheep farmers in Limpopo on the use of the FAMACHA system, to determine the correct timing of deworming. The use of the FECRT, EHT, and MALDT provided an identification of the occurrence of resistance against anthelmintics in Limpopo Province. Under–dosing, which could be due to lack of weighing equipment, and high treatment frequencies due to lack of proper training on anthelmintic use may have contributed in the findings of this study. The use of a heart girth measurement tape similar to those used in cattle and pigs is, therefore, recommended as this would certainly provide resource-poor farmers with a practical tool to use in determining the live weight of their small stock.

## Authors’ Contributions

MM, AMT, RM, and OMMT planned the study. MM conducted the experiments and drafted the manuscript. DMK generated the GIS maps. AMT, RM, DMK, and OMMT reviewed the manuscript. All authors have read and approved the final manuscript.
